# Effect of Isothermal Annealing on Sn Whisker Growth Behavior of Sn0.7Cu0.05Ni Solder Joint

**DOI:** 10.3390/ma16051852

**Published:** 2023-02-24

**Authors:** Aimi Noorliyana Hashim, Mohd Arif Anuar Mohd Salleh, Muhammad Mahyiddin Ramli, Mohd Mustafa Al Bakri Abdullah, Andrei Victor Sandu, Petrica Vizureanu, Ioan Gabriel Sandu

**Affiliations:** 1 Centre of Excellence Geopolymer and Green Technology, Universiti Malaysia Perlis (UniMAP), Taman Muhibah, Jejawi, Arau 02600, Perlis, Malaysia; 2 Faculty of Chemical Engineering and Technology, Universiti Malaysia Perlis (UniMAP), Arau 02600, Perlis, Malaysia; 3 School of Microelectronic Engineering, Pauh Putra Campus, Universiti Malaysia Perlis (UniMAP), Arau 02600, Perlis, Malaysia; 4 Faculty of Materials Science and Engineering, Gheorghe Asachi Technical University of lasi, Blvd. D. Mangeron 71, 700050 Iasi, Romania; 5 Romanian Inventors Forum, Str. Sf. P. Movila 3, 700089 Iasi, Romania; 6 National Institute for Research and Development in Environmental Protection INCDPM, Splaiul Independentei 294, 060031 Bucharest, Romania; 7 Technical Sciences Academy of Romania, Dacia Blvd 26, 030167 Bucharest, Romania

**Keywords:** Sn whisker, Sn0.7Cu0.05Ni, IMC interfacial, (Cu,Ni)_6_Sn_5_, solder joint, annealing, suppression, mitigation

## Abstract

This paper presents an assessment of the effect of isothermal annealing of Sn whisker growth behavior on the surface of Sn0.7Cu0.05Ni solder joints using the hot-dip soldering technique. Sn0.7Cu and Sn0.7Cu0.05Ni solder joints with a similar solder coating thickness was aged up to 600 h in room temperature and annealed under 50 °C and 105 °C conditions. Through the observations, the significant outcome was the suppressing effect of Sn0.7Cu0.05Ni on Sn whisker growth in terms of density and length reduction. The fast atomic diffusion of isothermal annealing consequently reduced the stress gradient of Sn whisker growth on the Sn0.7Cu0.05Ni solder joint. It was also established that the smaller (Cu,Ni)_6_Sn_5_ grain size and stability characteristic of hexagonal η-Cu_6_Sn_5_ considerably contribute to the residual stress diminished in the (Cu,Ni)_6_Sn_5_ IMC interfacial layer and are able to suppress the growth of Sn whiskers on the Sn0.7Cu0.05Ni solder joint. The findings of this study provide environmental acceptance with the aim of suppressing Sn whisker growth and upsurging the reliability of the Sn0.7Cu0.05Ni solder joint at the electronic-device-operation temperature.

## 1. Introduction

The main impetus of Sn whisker nucleation and growth is residual stress [1,2,3] by soldering. As a response over time, stresses release mechanism by diffusion [2,4,5] and compressive residual stress, and Sn whiskers are initiated and protrude out spontaneously at their base of substrate that may contribute immense apprehension to the electronics industry [1,6,7]. The major source of compressive stress in lead-free solder joints is intermetallic compound (IMC) interfacial evolution as per the continuous reaction of a Sn-rich solder with a copper (Cu) substrate forming intermetallic compounds within the grain boundaries [2,5,7,8,9,10,11]. The formation of the Cu_6_Sn_5_ layer by the IMC growth–diffusion gradient between 109 °C and 220 °C is an adequate and continuously controlled deliberate reaction process [1]. Illés et al. established the ability of spontaneous Sn whiskers to initiate and grow on surface of a solder joint with an average thickness of solder coating of 400 nm as deposited. They also provided evidence that the main stimulus of the whiskering intensity on the solder coating surface is induced by high compressive stress of IMC interfacial layer [2].

The properties of the lead-free solder joint between the Sn-rich solder and the Cu substrate affect the Sn whisker growth behavior of the surface. Horvath et al. concluded that the thickness of the solder coating and the grain size are the main properties accountable for Sn whisker behavior [12,13]. A thicker solder coating provides better stress relaxation capacity [2,14] and exhibits a lower inclination to whisker nucleation and growth. A thicker solder coating requires more incubation time for the interfacial IMC to diffuse and move up the grain boundaries to develop the occupied compressive stress cell [13]. Horváth et al. endorsed that the thickness of the solder coating for electronic devices should be 8 μm at minimum in order to suppress the vulnerability of Sn whisker growth, as well to prolong incubation time [13]. The effect of solder grain size also correspondingly contributes stress that is induced in solder coating. To correlate the atomic diffusion of the grain boundary and the formation of the IMC interfacial layer, it is accepted that more stress is produced during grain boundary diffusion of smaller grains. However, Sn whiskers proceed with lower stress for the purpose of grain boundary sliding for nucleation and growth in small grain boundary diffusion. As an outcome, the resistance of smaller grains is improved against the low residual stress gradient on behalf of the stress relaxation of the solder coating layer [13]. In a previous study, Hashim et al. found that Sn0.7Cu0.05Ni with a smaller grain size of (Cu,Ni)_6_Sn_5_ had a lower inclination to Sn whisker propensity compared to the Sn0.7Cu solder joint [14].

Numerous studies of mitigation methods have been suggested to suppress Sn whisker formation. The conformal coating or the added-nickel (Ni) under-layer between the solder and the Cu substrate is a well-known method used to mitigate Sn whisker formation [10], which has comparable outcomes to avoiding large grain growth of the IMC interfacial layer between a Sn-rich solder and a Cu substrate. Distinctly, these approaches are significant in terms of temporary effect as, in many cases, Sn whiskers are able to penetrate conformal coatings. The findings of these reference studies are accepted in terms of delayed incubation time for Sn whisker growth [3,10]. Isothermal annealing is also one of the common methods used to mitigate the Sn whisker by developing a fine and uniform IMC interfacial layer between the Sn-rich solder and the Cu substrate. This IMC interfacial layer inhibits the formation of a large grain in the IMC layer by promoting fast diffusion of Cu grain boundaries in the Sn-rich layer and yielding the relief of internal stresses in the solder joint [8,13]. 

In the view of the constrained temperature range for the nucleation and growth of Sn whiskers, there has been limited significant research on how isothermal annealing affects Sn whisker behavior [15]. When the temperature is too low, slow atomic diffusion, takes place, owing to the insufficient kinetics. Furthermore, when the temperature is too high, fast atomic diffusion occurs; therefore, there is not enough of a driving gradient because of stress release [8]. As stated in previous research, isothermal annealing at 150 °C and aged for 30 min is a representative technique used for the inhibition of Sn whisker growth at ambient temperatures. Fukuda et al. conducted a reduction assessment of whisker density periodically over 8 months with isothermal annealing at 50 °C [7]. They also detected a reduction in maximum Sn whisker length with isothermal annealing at 150 °C and ageing for 1 h. In addition, Kim et al. also asserts that annealing at 125 °C is useful to the inhibition of Sn whisker growth without the growth of Cu_3_Sn [13].

The purpose of this study was to analyze the effects of isothermal annealing on Sn whisker growth behavior of the Sn0.7Cu0.05Ni solder joint using the hot-dip soldering technique. Isothermal annealing at 50 °C and 105 °C with an ageing time of up to 600 h pointedly represents an accelerated test to obtain life extrapolation at the electronic-device-operation temperature. To evaluate the practicality of the mitigation strategies of Sn whiskers, the Sn0.7Cu solder joint was used as a point of reference.

## 2. Materials and Methods

### 2.1. Materials and Sample Preparation

The lead-free solder alloys of Sn-0.7Cu and Sn-0.7Cu-0.05Ni were provided by Nihon Superior Co., Ltd. in Osaka, Japan. For sample preparation, the high-purity Cu substrate (1.5 cm × 1.5 cm × 0.1 cm) was cleaned with a 5% hydrochloric acid solution and deionized water for 3 min to remove surface oxides, then it was rinsed with acetone followed by distilled water, and subsequently air-dried to dispose of oil impurity. Then, the Cu substrate was dipped in a standard halogen rosin-activated flux solution of the Japanese Industrial Standard, JIS Z3198-4, in order to seal out air and improve soldering wetting characteristics.

### 2.2. Hot-Dip Soldering 

**Figure 1:**
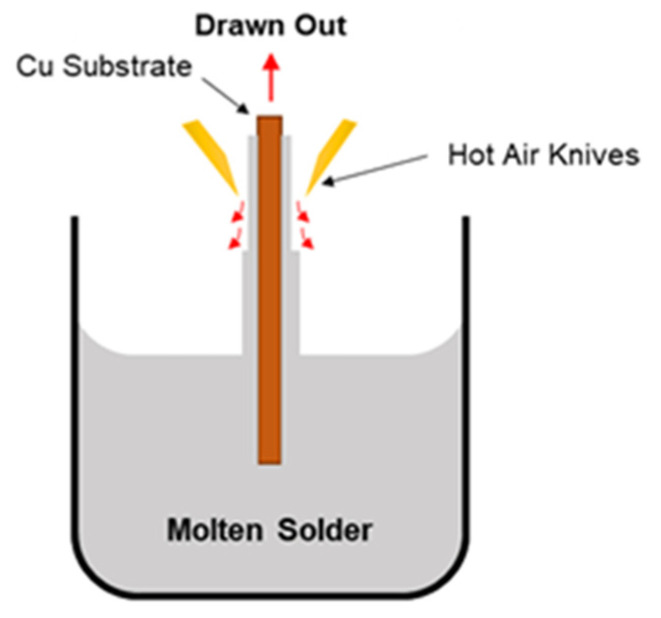
Schematic diagram of a hot-dip soldering process.

The Sn-0.7Cu and Sn-0.7Cu-0.05Ni solder joint was synthesized using the hot-dip soldering technique. This technique provides well-controllable lead-free solder coating thickness parameters. During the hot-dip soldering process, the molten solder pot was heated up to 265 °C and the Cu substrate was placed between a pair of blower air knives. The Cu substrate was immersed in molten solder for 2 s and withdrawn at a speed of 10 mm/s. During withdraw, the air knives system wiped down the excessive solder (tinning process) adhered on both sides of the substrate surface by impinging the pressured hot air as shown in Figure 1. The controllable hot air of the knives was used to control the thickness of the total solder coating and the uniformity of the coating, particularly near the substrate edge. The pressure of hot air comprised the range 0.1 MPa to 1.0 MPa to obtain the condition of comparable solder coating thickness for Sn whisker growth evaluation. The samples were then washed with acetone and distilled water after cooling for 30 min. In order to saturate the internal residue stress induced by Cu_6_Sn_5_ IMC formation, samples were aged for 48 h at room temperature.

### 2.3. Testing and Characterization 

The samples of the Sn0.7Cu and Sn0.7Cu-0.05Ni solder joint was investigated by performing isothermal annealing at 50 °C and 105 °C for ageing times 0, 100, 200, 300, 400, 500, and 600 h. The samples were annealed in a mechanical-convection heating oven provided by Thermo Scientific. For reference assessment, the samples were observed at room temperature with the same ageing time (0–600 h).

**Figure 2:**
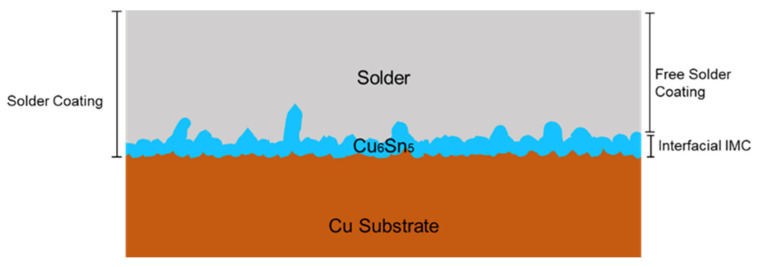
The thickness of measuring solder coating, free solder thickness, and Cu_6_Sn_5_ IMC interfacial layer.

The metallography surface and cross-section of the solder joint morphology of Sn whisker behavior were found using the secondary and backscattered electron imaging approaches of a scanning electron microscope, SEM (JEOL-JSM-6010LA), JEOL Ltd., Tokyo, Japan. The samples were deep-etched using 2% 2-nitrophenol, 5% sodium hydroxide solution, and 93% distilled water for a top view of the Cu_6_Sn_5_ IMC interfacial layer. Figure 2 presents the illustrative cross-sectional schematic of the solder joint. A measured average value of five interpretations of the thickness value of the solder coating, the IMC interfacial layer, and particle size analyses were examined using Image-J programmed software (1.8.0 open source software) as specified in previous research [14]. 

**Figure 3:**
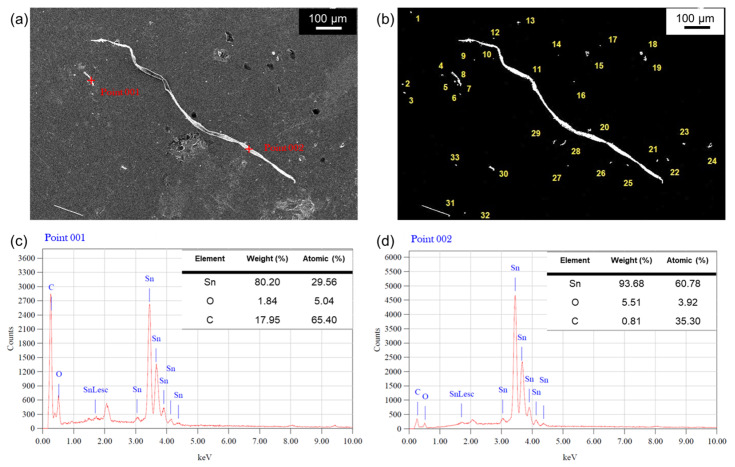
The method of image processing adapted to calculate density of Sn whisker growth and element analyses of Sn whisker (**a**) SEM-EDX grayscale image, (**b**) binary thresholding segmentation image, (**c**) EDX of point 001, (**d**) EDX of point 002.

The statistic evaluation of Sn whisker behavior was measured based on the whisker standards (JESD22-A121A) of the Joint Electron Device Engineering Council (JEDEC). The analyses of the length and density distribution of the Sn whisker were structured with ±5% accuracy and five preference average values of interpretations analyses using Image-J Software. In order to attain a statistically consistent assessment, the density of Sn Whiskers were analyzed using the binary thresholding image-segmentation technique. This technique separates the foreground pixels from the background pixels and produces binary segmentation images from Scanning electron microscopy (SEM) grayscale images as illustrated in Figure 3a,b. Respectively, Figure 3c,d indicate the validation of the elemental Sn whisker assessment using SEM-EDX analyses.

For wettability analyses, the samples of the Sn0.7Cu and Sn0.7Cu0.05Ni solder were rolled and punched into a diameter of 0.2 mm, then reflowed onto the Cu substrates. The reflow time was conducted at 250 °C for 127 s. The samples were mounted, carried out using an optical microscope (OM) and Image-J software for wettability properties. This investigation was attained by measuring the wetting degree of the contact angle between the Sn-rich solder and the Cu substrate. The thermodynamic assessment of the phase diagram, phase equilibrium, and phase transformation was validated using the Calphad method and Thermo-Calc-2021a database TCSLD v3. 3. A binary crystal structure phase diagram of Sn-Cu and Sn-Ni preferred to justify the phase transformation of Sn0.7Cu and Sn0.7Cu0.05Ni evidently.

## 3. Results and Discussion

**Figure 4:**
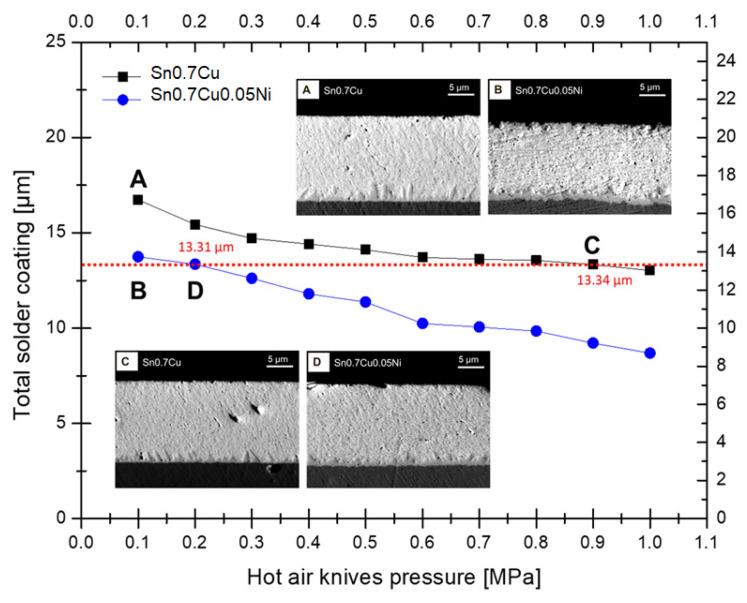
The solder coating thickness of Sn0.7Cu and Sn0.7Cu0.05Ni with hot air knives’ pressure.

It should be noted that Sn whisker nucleation and growth is significantly correlated to total solder coating thickness. A thicker solder coating resulted in more residual stress and incubation time of Sn whisker growth on the solder surface [2,14,15,16,17,18,19,20]. Figure 4 shows a thickness observation of the total solder coating after hot-dip soldering with different pressures of hot air knives. The data clearly show the conditions of the equivalent solder coating thickness for Sn0.7Cu and Sn0.7Cu0.05Ni in the evaluation of Sn whisker growth. The total solder thickness decreased proportionally to the hot air knives’ pressure, as well as dissimilar thicknesses with different type of solder. In order to obtain comparable solder thicknesses for both Sn0.7Cu and Sn0.7Cu0.05Ni, the hot air pressure was in the wide range of 0.1 to 10 MPa. At the lowest pressure of the hot air knives, 0.1 MPa, the total solder coating of Sn0.7Cu was 16.74 µm (A), and Sn0.7Cu0.05Ni was 13.82 (B) µm. Meanwhile, at the highest pressure of 10 MPa, the total solder coating of Sn0.7Cu was 13.20 µm and 8.68 µm for Sn0.7Cu0.05Ni. The similar total thickness coating of Sn0.7Cu was 13.34 µm (C) at 0.9 MPa, and Sn0.7Cu0.05Ni was 13.31 µm (D) at 0.2 MPa.

**Figure 5:**
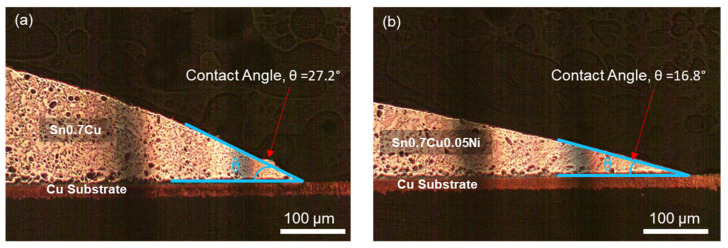
The wetting contact angle of solder joint (**a**) Sn0.7Cu, (**b**) Sn0.7Cu0.05Ni.

This consequence noticeably shows the purpose of hot air knives in controlling the thickness of the total solder coating. Additionally, the variation in total solder thickness significantly indicates the solder wettability properties of Sn0.7Cu and Sn0.7Cu0.05Ni. The wettability of the solder was evaluated through the wetting angle of the solder joint as presented in Figure 5. A larger wetting contact angle allows for a higher-thickness solder coating to be completed. The Sn0.7Cu0.05Ni solder joint showed better wettability with a smaller contact angle, θ = 16.8°, related to the Sn0.7Cu solder joint with contact angle θ = 27.2°. The superior fluidity properties of the Sn0.7Cu0.05Ni solder alloy are capable of producing reliable in the uniformity of the thickness of the Cu substrate. As agreed by Gain et al., Ni alloying enhances wettability, which may influence the mechanical reliability of the interconnection [16]. The optimum wettability that relates to lower surface-interfacial energy could be achieved by minimizing the contact angle value [17].

**Figure 6:**
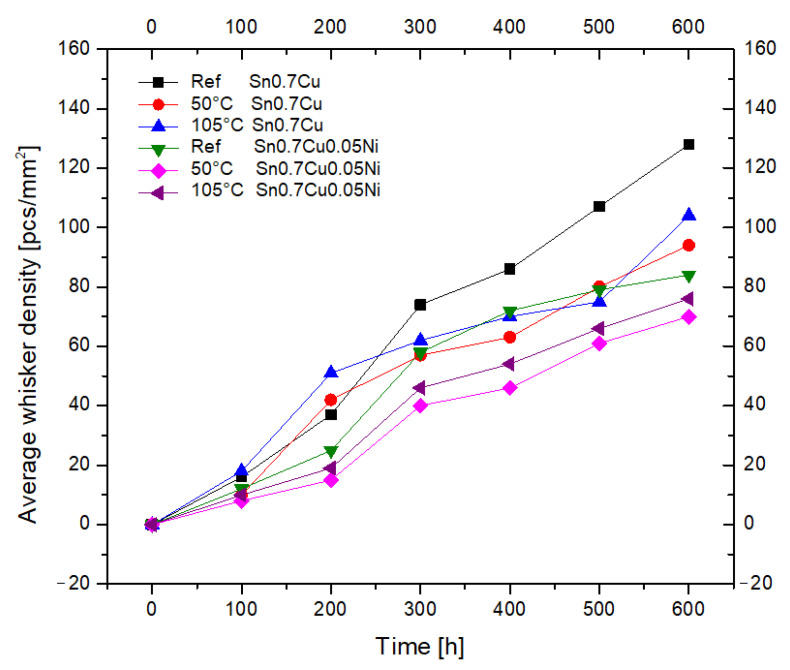
Average Sn whiskers density with isothermal ageing.

Figure 6 shows the average Sn whisker densities of the Sn0.7Cu and Sn0.7Cu0.05Ni solder joints with an average total solder thicknesses ±13.3 µm, ageing up to 600 h in room temperature, and annealed under 50 °C and 105 °C conditions. From the observation, most of the Sn whiskers were grown and noticed after 100 h of ageing under all conditions. After ageing up to 600 h at room temperature, the reference sample showed that the density of the Sn whisker reached 128 pcs/mm^2^ on the Sn0.7Cu solder joint and 84 pcs/mm^2^ on the Sn0.7Cu0.05Ni solder joint. Additionally, for the Sn0.7Cu and Sn0.7Cu0.05Ni solder joints, the observation also showed an increased growth in the Sn whiskers with annealed ageing time. Additionally, the density of Sn whisker growth indicated a reduction trend in the increase in isothermal annealing temperature. It is interesting to point out that the intensity of Sn whisker formation is higher at Sn0.7Cu related to the Sn0.7Cu0.05Ni solder joint.

**Figure 7:**
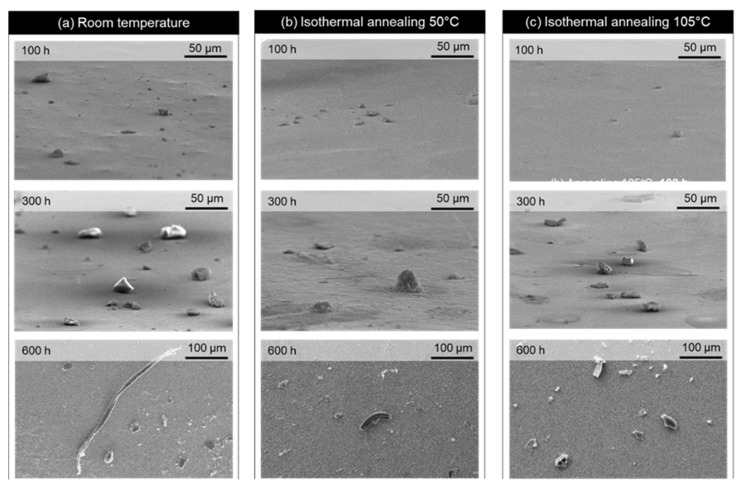
Surface morphology of Sn whisker growth on Sn0.7Cu0.05Ni aged at 100 h, 300 h, and 600 h with isothermal ageing at (**a**) room temperature, (**b**) 50 °C, and (**c**) 105 °C.

Figure 7 shows the surface morphology of Sn whisker nucleation and growth on Sn0.7Cu0.05Ni at ageing times 100 h, 300 h, and 600 h with room temperature ageing, 50 °C isothermal ageing, and 105 °C isothermal ageing. The number of Sn whiskers on the Sn0.7Cu0.05Ni solder joint with isothermal ageing increased scattering with ageing duration. Furthermore, the intensity of Sn whisker growth increased with ageing at room temperature of the reference sample, compared to the annealed samples under 50 °C and 105 °C conditions. After 100 h ageing time, the scattered nucleation of Sn whiskers, particularly under room temperature conditions, can be observed. It can also be observed that Sn whiskers significantly intensify to initiate after 300 h of ageing.

The length of the Sn whiskers after 600 h of ageing time at room temperature was obviously longer and had a greater density of Sn whisker growth per unit area. However, in the case of the annealed samples, the 105 °C condition increased the density of Sn whisker growth compared to the annealed samples 50 °C. It can be determined that isothermal annealing is functional for Sn whisker mitigation. The significant outcome was detected through the observation of the suppressing effect of Sn0.7Cu0.05Ni on Sn whisker growth in terms of density and length reduction.

**Figure 8:**
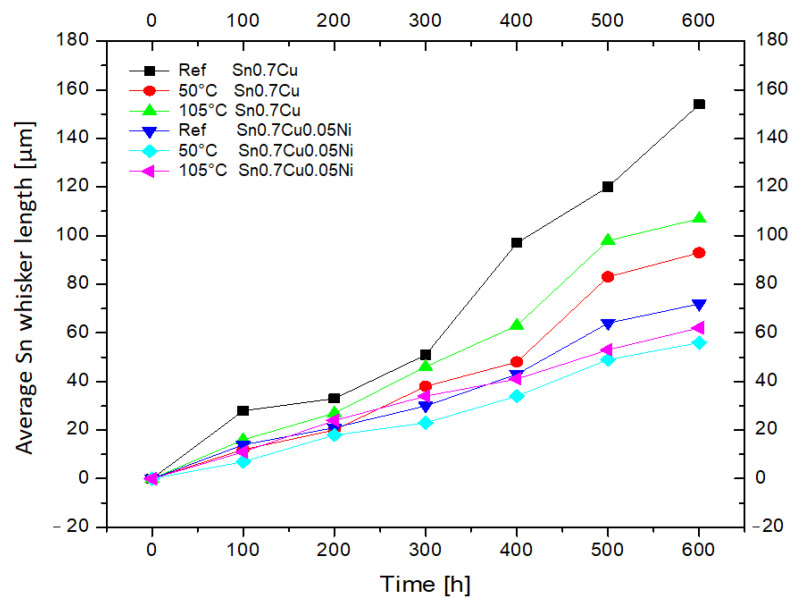
Average Sn whisker length with isothermal ageing.

The average Sn whisker lengths measured indicate that isothermal annealing is effective at reducing whisker lengths as observed in Figure 8. The length of the Sn whiskers increased with increasing ageing time and annealing temperature. The condition where the annealing temperature was elevated from 50 °C to 105 °C had a small length-reducing effect. This decrease effect was similar for both the annealed Sn0.7Cu and Sn0.7Cu0.05Ni solder joint samples. It was reported by Kim et al. that the behavior of Sn whiskers after 50 °C isothermal annealing for 6-month ageing is similar to Sn whisker growth in ambient storage for a 1-year-ageing duration in terms of size and of shape [21]. It is also noteworthy that Sn0.7Cu0.05Ni was seen to reduce the length of the Sn whiskers compared to Sn0.7Cu, either in room temperature or annealing temperature. This further confirms that Sn0.7Cu0.05Ni has a suppression effect in retarding the length of Sn whiskers.

**Figure 9:**
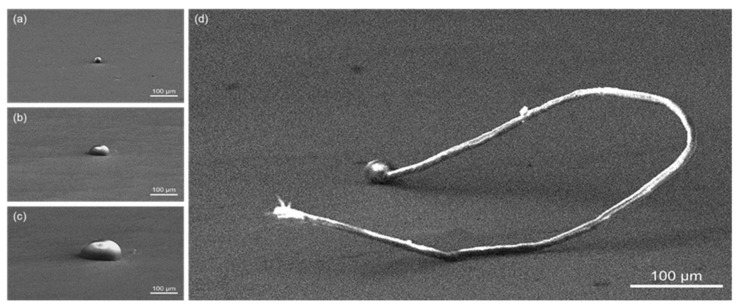
A quantitative evaluation of Sn whisker growth on the surface of the Sn0.7Cu solder joint with isothermal annealing at 50 °C for (**a**)100 h; (**b**) 200 h; (**c**) 400; and (**d**) 600 h.

Figure 9 identifies the quantitative assessment of nucleation and growth from a small nodule to the longest-length 763 µm Sn whiskers assessed from the surface of the Sn0.7Cu solder coating with 50 °C isothermal annealing and up to 600 h ageing time. The assessment of the kinetic growth of Sn whiskers are relative to the stress gradients induced in the solder coating layer [5]. Moreover, it can be clearly observed in Figure 9d that the conductive Sn whiskers able to grow longer may lead to reliability apprehension in miniaturization trends in the electronics industry [22]. This finding did not certainly occur toward the end of the observation as long Sn whiskers have a tendency to break off impulsively [13]. Additionally, Tu et al. validated that it is possible to short circuit as the Sn whiskers could break and fall between two neighboring conductors [8].

**Figure 10:**
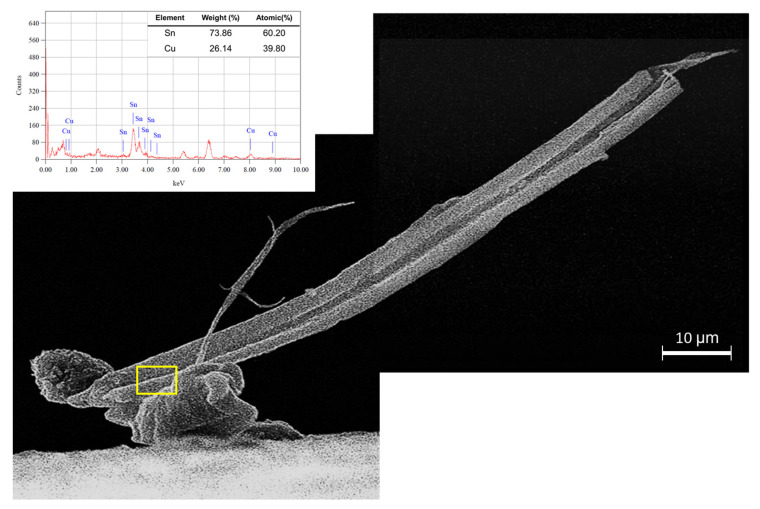
SEM-EDX analyses of kinked Sn whisker growing from nodule on Sn0.7Cu solder joint with isothermal annealing at 105 °C for 400 h.

**Figure 11:**
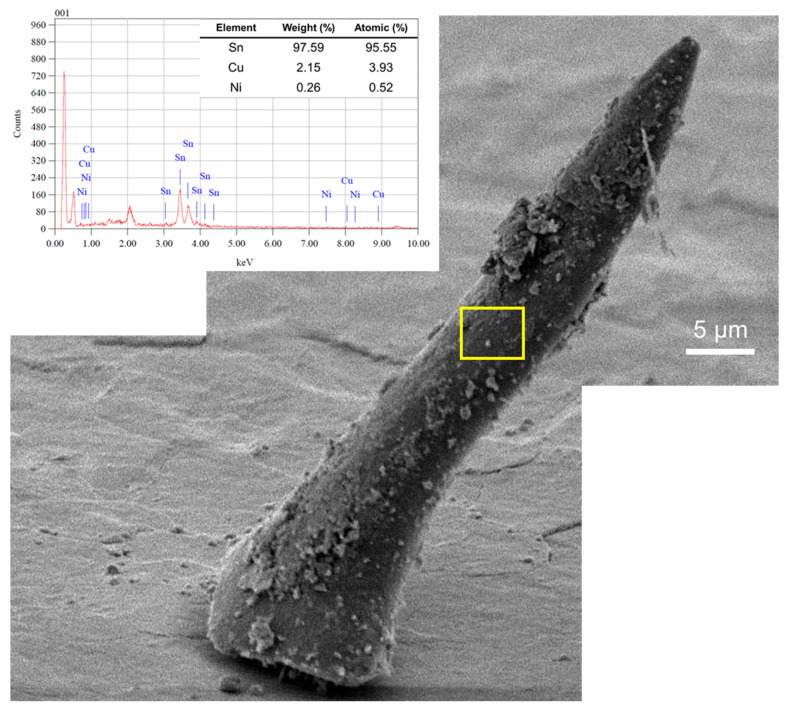
SEM-EDX analyses of faceted Sn whisker on Sn0.7Cu0.05Ni solder joint with isothermal annealing at 105 °C for 400 h.

Figure 10 and Figure 11 show the comparison between Sn whisker growth behavior on the Sn0.7Cu and Sn0.7Cu0.05Ni solder joints with isothermal annealing at 105 °C for 400 h. Sn whiskers of the long filament with a very high aspect ratio were commonly observed on the surface of the Sn0.7Cu solder joint, whereas the short Sn whiskers or hillocks were spotted on the surface of Sn0.7Cu0.05Ni solder joint. Figure 10 presents kinked Sn whiskers growing from the nodule with fine striation marks along the length of the outer surface. The Sn whisker displays fine striations along the whisker axis grooves along their length that are characteristic of typical Sn whiskers seen in the literature, as Sn whiskers are extruded and tend to propagate from the weakest points of grain boundaries [21] through surface cracks and imperfection sites.

Figure 11 shows the evaluation results of Sn whisker growth behavior on the Sn0.7Cu0.05Ni solder joint. However, a small composition of 0.05% Ni added to the Sn0.7Cu solder prompted a significant suppression effect on Sn whisker aspect-ratio growth. It was perceived that the Sn whiskers became denser and shorter than Sn whiskers on the Sn0.7Cu solder joint. The rate of Sn whisker growth on the Sn0.7Cu0.05Ni solder joint was much slower than that of the Sn0.7Cu solder joint with a divergent aspect ratio and morphology.

The formation of the Cu_6_Sn_5_ IMC interfacial layer between the Cu substrate and the Sn-rich solder was closely related to the Sn whisker growth behavior. In view of the formation of the Cu_6_Sn_5_ IMC interfacial layer as a main stimulus of the compressive stress in the solder coating layer, the effect of isothermal annealing had to be considered a substantial reliability aspect of the Sn whisker acceleration factor. An isothermal-annealing accelerated test provided the thermal kinetic mechanism of a lifetime at the device operation temperature.

**Figure 12:**
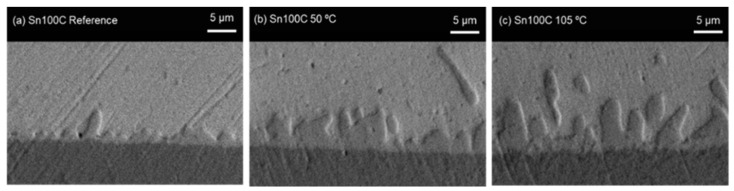
An assessment of the (Cu,Ni)_6_Sn_5_ IMC interfacial layer on the Sn0.7Cu0.05Ni solder joint after ageing for 300 h at (**a**) room temperature, (**b**) isothermal annealing 50 °C, and (**c**) isothermal annealing 105 °C.

Figure 12 presents the growth revolution of the thickness morphology of the (Cu,Ni)_6_Sn_5_ IMC interfacial layer on the Sn0.7Cu0.05Ni solder joint after isothermal ageing for 300 h at room temperature, annealed at 50 °C and 105 °C. It was revealed that the thickness of the (Cu,Ni)_6_Sn_5_ IMC interfacial layer was upsurged and uniform relative to the isothermal ageing temperature and duration. The average thickness of the (Cu,Ni)_6_Sn_5_ IMC interfacial layer aged for 300 h at room temperature was 1.34 µm and increased to 3.52 µm after annealing at 50 °C, before increasing to 5.48 µm after annealing at 105 °C. It proposes that Cu atoms diffuse more intensely through higher activation energy in the Sn solder layer at the isothermal annealing temperature. The activation energy and growth rate of the (Cu,Ni)_6_Sn_5_ IMC interfacial layer on the Sn0.7Cu0.05Ni solder joint were stimulated by the isothermal annealing temperature. At 105 °C, the growth of the (Cu,Ni)_6_Sn_5_ IMC interfacial layer with a higher Cu diffusion rate was faster compared to being annealed at 50 °C. Additionally, the uniform and stable IMC interfacial layers could be protective layers for Sn whisker nucleation from the solder joint [15]. In addition, isothermal annealing possibly significantly decelerates the irregular growth of Cu_6_Sn_5_ IMC interfacial layer against bulk diffusion and reduce lattices imperfections thus diminish residual stress in solder coating [15].

In correlation with the stress generation due to the formation of the IMC interfacial layer and the growth rate of the Sn whiskers, it is supposed that the stress is relieved in sequence to the fast atomic diffusion of the IMC interfacial layer [8], and therefore reduces the stress gradient of Sn whisker growth. In this study, the formation of the Cu_3_Sn IMC interfacial layer was not obviously apparent, possibly because Sn0.7Cu0.05Ni suppressed the formation of the Cu_3_Sn IMC interfacial layer [23,24]. It was observed that the formation of the Cu_6_Sn_5_ and Cu_3_Sn IMC interfacial layer during isothermal annealing was able to be function as a continuous diffusion barrier and was thus less prone to whisker formation [8,15].

**Figure 13:**
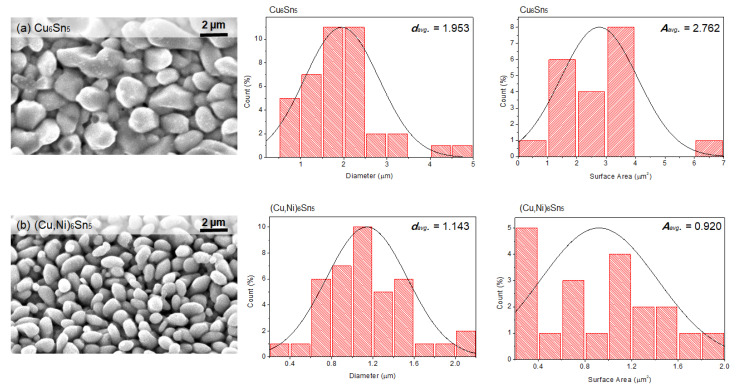
Morphology and particle size analyses of IMC interfacial layer after aging for 300 h at 50 °C: (**a**) Cu_6_Sn_5_ and (**b**) (Cu,Ni)_6_Sn_5_.

It is marked that the grain structure of the IMC interfacial layer also intensely regulates the behavior of Sn whisker growth. Figure 13 show the microstructure and particle size analyses of the Cu_6_Sn_5_ and (Cu,Ni)_6_Sn_5_ IMC interfacial layer after isothermal ageing for 300 h at 50 °C. It is remarkable that the average grain size of (Cu,Ni)_6_Sn_5_ was 1.143 µm, which is considerably smaller than average grain size of Cu_6_Sn_5_ 1.953 µm. It was established by Horvath et al. that a larger grain size induced more susceptibility to Sn whisker growth. The smaller grain sizes result in smaller grain boundary areas, which is a consequence for Sn whisker grains as it takes less stress to cause grain boundary sliding. Thus, the Sn0.7Cu solder joint is more vulnerable to whiskering than Sn0.7Cu0.05Ni [13].

**Figure 14:**
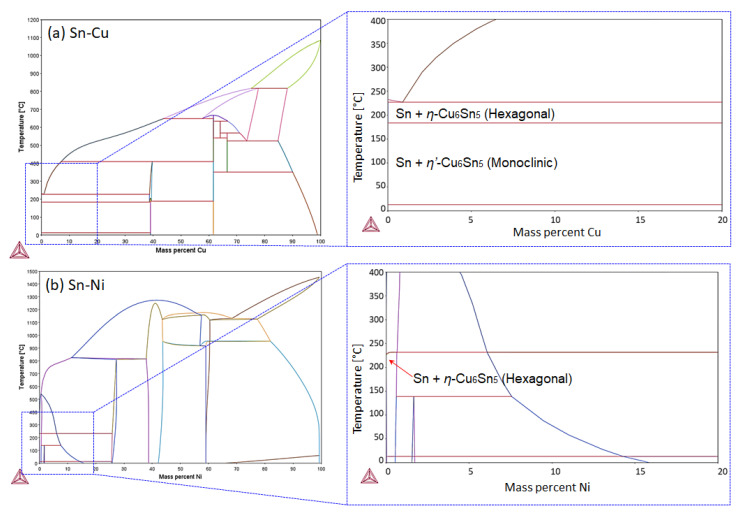
The binary phase diagram of (**a**) Sn-Cu system and (**b**) Sn-Ni system, simulated using Calphad method, Thermo-Calc-2021a database TCSLD v3.3.

The accompanying aspect that inclines the invulnerable growth of the Sn whisker in the Sn0.7Cu0.05Ni solder joint is the phase stabilization of the IMC interfacial layer. Figure 14 shows a summary of the binary phase equilibrium for the Sn-Cu and Sn-Ni system using Thermo-Calc software. It presents the allotropic transformation of Cu_6_Sn_5_ IMC, occurring at a temperature that falls approximately below 186 °C, which causes a structural change from hexagonal η-Cu_6_Sn_5_ to monoclinic η’-Cu_6_Sn_5_ [24]. Meanwhile, the (Cu,Ni)_6_Sn_5_ IMC maintains stability in the hexagonal η-Cu_6_Sn_5_ phase. The stabilizing effect of the crystal structure the during cooling process in the hexagonal η-Cu_6_Sn_5_ phase inhibits volume modifications that may possibly come up with the residual stress diminished in the IMC interfacial layer [14,25,26]. The thermal reduction difference in the Sn solder and the substrate for the duration of the cooling process from the deposition soldering temperature correspondingly contributes to Sn whisker formation. In addition, the transformation of volume expansion in the intermetallic layer is significant to the properties of lower density of the IMC interfacial layer related to the Cu substrate. The volume expansion also produces compressive stresses of the Cu and Sn interface in the vertical direction against Sn solder coating [2]. Therefore, it is suggested that the stability characteristic of the (Cu,Ni)_6_Sn_5_ IMC is able to suppress Sn whisker growth on the Sn0.7Cu0.05Ni solder joint [14].

## 4. Conclusions

The assessment of Sn whisker growth behavior on the surface of the Sn0.7Cu0.05Ni solder joint using the hot-dip soldering technique was examined after ageing to 600 h at room temperature, annealed under 50 °C and 105 °C conditions. The obtained conclusions are as follows:

(i) The Sn whisker growth on the Sn0.7Cu0.05Ni solder joint was significantly mitigated with isothermal annealing.
(ii) Isothermal annealing stimulates the activation energy and faster atomic diffusion of the IMC interfacial layer and hence reduces the stress gradient for Sn whisker growth on the Sn0.7Cu0.05Ni solder joint.
(iii) The stability characteristic of hexagonal η-Cu_6_Sn_5_ significantly contributes to the residual stress diminished in the IMC interfacial layer of (Cu,Ni)_6_Sn_5_ IMC and is able to suppress the formation and growth of Sn whiskers on the Sn0.7Cu0.05Ni solder joint.

The findings of this study provide environmental acceptance with the aim of suppressing Sn whisker growth and upsurging the reliability of the Sn0.7Cu0.05Ni solder joint at electronic-device-operation temperature.

## Data Availability

Not applicable.
